# Low Energy Shock Wave Therapy Induces Angiogenesis in Acute Hind-Limb Ischemia via VEGF Receptor 2 Phosphorylation

**DOI:** 10.1371/journal.pone.0103982

**Published:** 2014-08-05

**Authors:** Johannes Holfeld, Can Tepeköylü, Stefan Blunder, Daniela Lobenwein, Elke Kirchmair, Marion Dietl, Radoslaw Kozaryn, Daniela Lener, Markus Theurl, Patrick Paulus, Rudolf Kirchmair, Michael Grimm

**Affiliations:** 1 University Hospital for Cardiac Surgery, Innsbruck Medical University, Innsbruck, Austria; 2 Division of Clinical and Functional Anatomy, Department of Anatomy, Histology and Embryology, Innsbruck Medical University, Innsbruck, Austria; 3 University Hospital for Dermatology and Venerology, Innsbruck Medical University, Innsbruck, Austria; 4 University Hospital for Internal Medicine III, Department of Cardiology and Angiology, Innsbruck Medical University, Innsbruck, Austria; 5 Clinic of Anaesthesiology, Intensive Care Medicine and Pain Therapy, Goethe-University Hospital, Frankfurt am Main, Germany; Cardiological Center Monzino, Italy

## Abstract

**Objectives:**

Low energy shock waves have been shown to induce angiogenesis, improve left ventricular ejection fraction and decrease angina symptoms in patients suffering from chronic ischemic heart disease. Whether there is as well an effect in acute ischemia was not yet investigated.

**Methods:**

Hind-limb ischemia was induced in 10–12 weeks old male C57/Bl6 wild-type mice by excision of the left femoral artery. Animals were randomly divided in a treatment group (SWT, 300 shock waves at 0.1 mJ/mm^2^, 5 Hz) and untreated controls (CTR), n = 10 per group. The treatment group received shock wave therapy immediately after surgery.

**Results:**

Higher gene expression and protein levels of angiogenic factors VEGF-A and PlGF, as well as their receptors Flt-1 and KDR have been found. This resulted in significantly more vessels per high-power field in SWT compared to controls. Improvement of blood perfusion in treatment animals was confirmed by laser Doppler perfusion imaging. Receptor tyrosine kinase profiler revealed significant phosphorylation of VEGF receptor 2 as an underlying mechanism of action. The effect of VEGF signaling was abolished upon incubation with a VEGFR2 inhibitor indicating that the effect is indeed VEGFR 2 dependent.

**Conclusions:**

Low energy shock wave treatment induces angiogenesis in acute ischemia via VEGF receptor 2 stimulation and shows the same promising effects as known from chronic myocardial ischemia. It may therefore develop as an adjunct to the treatment armentarium of acute muscle ischemia in limbs and myocardium.

## Introduction

Peripheral and coronary vascular disease still represent a major socio-economic health burden in industrialized countries. The effect of surgical or interventional revascularization is limited due to nonviable myocytes. Therefore clinicians are still in need of regenerative therapies as an adjunct to their treatment armentarium. Current treatment strategies for regeneration of infarcted muscle include (stem) cell or gene therapy based approaches. However, none of them yet gained broad clinical use due to distinct limitations [1]. Although shown beneficial in numerous pre-clinical and clinical trials some challenges remain. In particular, the ideal cell type, the way of cell administration and homing of cells are still a matter of research in stem cell therapy [2]–[4]. Gene therapy with different kind of vectors as well shows promising results, however delivery approaches and expression regulation of therapeutic gene products remain challenging [5], [34].

A clinically safe and feasible regenerative therapy could dramatically improve outcome of patients as well as their long-term quality of life. Shock wave treatment is used in medicine for over 30 years for lithotripsy [6]. At lower energy levels it was shown to induce regeneration in bone and soft tissue without any side effects. In orthopedics and traumatology shock waves are therefore successfully used in clinical routine for regeneration of non-healing bones and wounds as well as for the treatment of tendinopathies such as the so-called “tennis elbow” [7]–[9].

Furthermore, low energy shock wave treatment (SWT) has been shown to cause relief of angina symptoms in patients suffering from coronary artery disease [10]–[12]. As a morphological correlate to this clinical effect the induction of angiogenesis has been shown in animal models of chronic myocardial ischemia [13]. Up-regulation of the pivotal angiogenic growth factors VEGF (vascular endothelial growth factor) and PlGF (placental growth factor) as well as their corresponding receptors Flt-1 (Fms-related tyrosine kinase 1, VEGFR 1) and KDR (kinase insert domain receptor, VEGFR 2) have been found. As a consequence left ventricular ejection fraction improved significantly [13]–[15].

In a recent work we have been able to proof that not only sprouting of existing vessels (angiogenesis), but as well the recruitment of endothelial progenitor cells for *de novo* vessel formation (vasculogenesis) via the SDF-1/CXCR4 axis is involved in neovascularization after shock wave treatment of ischemic muscle [16]. However, the limitation of regenerating infarcted muscle is represented by the infarction scar. Shock waves as well as all other regenerative approaches aim to recruit hibernating myocytes in order to strengthen the infarction border zone, thereby leading to functional improvement. Full functional restoration is not yet possible [17], [18].

Shock wave therapy is developing for a large variety of indications, including acute and chronic soft tissue wounds [19]–[21]. Results such as the induction of angiogenesis seem to be independent from the time point of treatment [21].

Having been shown to induce angiogenesis and vasculogenesis in chronic ischemic muscle, there is still no knowledge about the effect of shock waves on acute muscle ischemia. However, patients presenting with acute infarction are a major group in the clinical setting. Shock wave treatment as an adjunct to state-of-the-art revascularization in acute limb ischemia or myocardial infarction could dramatically improve outcome and quality of life if the effects were comparable to those seen in the chronic setting.

The present experiments therefore were intended to proof whether shock wave treatment induces angiogenesis in acute ischemia and to evaluate a stable long-term benefit resulting in improvement of blood perfusion.

## Materials and Methods

### Animals

The experiments were approved by the institutional animal care and use committee at Innsbruck Medical University and by the Austrian ministry of science (BMWF-66.011/0153-II/3b/2012). The investigation conformed to the “Guide for the Care and Use of Laboratory Animals” published by the US National Institutes of Health (NIH Publication No. 85–23, revised 1996; available from: www.nap.edu/catalog/5140.html).

Male adult C57/BL6 mice (Charles River, Sulzfeld, Germany) weighing 25–30 g were randomly divided into 4 groups (n = 10). Treatment animals (SWT) received shock wave therapy immediately after hind limb ischemia induction. Control animals (CTR) were treated equally without receiving treatment. The animals were sacrificed 72 h and 28 days after therapy.

### Hind limb ischemia

Anesthesia was administered by an intraperitoneal injection of ketamine hydrochloride (Graeub, Switzerland; 80 mg/kg body weight) and xylazine hydrochloride (aniMedica, Germany; 5 mg/kg body weight). Left femoral artery was ligated and excised between the inguinal ligament and proximal to the branching into saphenous and popliteal artery using 7-0 polypropylene sutures (Ethicon, USA). For analysis, the whole adductor muscle was harvested and split in half resulting in a proximal and distal portion. Of the distal portion, the middle part around the former femoral artery was used for analysis to avoid sampling variances of regions with greater or lesser ischemia.

### Shock wave treatment

Treatment was applied as described elsewhere [7]. Briefly, animals received shock wave therapy immediately after excision of the femoral vessels and skin closure, still under anesthesia at the area above the adductor muscles. Common ultrasound gel was used for coupling. The commercially available Orthogold device with applicator CG050-P (TRT LLC, Tissue Regeneration Technologies, Woodstock, GA, USA) served as shock wave device. The diameter of the applicator’s membrane is 4.5 centimeters. 300 impulses were delivered to the ischemic area with an energy flux density of 0,1 mJ/mm^2^ at a frequency of 5 Hz. The overall treatment time was about 3 minutes. The rationale or the treatment parameters is our experience from previous studies [16], [18]. At this low energy level no adverse effects were observed.

### RNA Isolation and quantitative Real Time (qRT) – PCR

RNA was isolated from muscle tissue using RNeasy Mini Kit (Quiagen, Valencia, CA, USA) according to the manufacturers instruction. RNA integrity was evaluated with agarose gel electrophoresis and RNA quantity was determined by spectrophotometry. Thereafter, one microgram of RNA was reverse transcribed using the IScript cDNA Synthesis Kit (BioRad, Hercules, CA, USA) and till further use stored at −20°C. All qRT-PCR were carried out on a BioRad iQ5 Cycler (BioRad Hercules, CA, USA) as described previously [22]. To control for variations in RNA quality and quantity, gene of interest (GOI) expression levels were normalized to the expression of β-actin. Relative Expression levels were calculated according to the formula: 2^−ΔCT^, where ΔCT was defined as CT (GOI) – CT (β-actin).

The following primer sequences were found using the Harvard’s PRIMER BANK website: β-actin, forward: 5′-GGCTGTATTCCCCTCCATCG-3′, reverse: 5′-CCAGTTGGTAACAATGCCATGT-3′; vascular endothelial growth factor (VEGF) - A, forward: 5′-GCACATAGAGAGAATGAGCTTCC-3′, reverse: 5′- CTCCGCTCTGAACAAGGCT-3′; Placental Growth Factor (PGF), forward: 5′-TCTGCTGGGAACAACTCAACA-3′, reverse: 5′- GTGAGACACCTCATCAGGGTAT-3′; FMS-like tyrosine kinase 1 (Flt1), forward: 5′- TGGCTCTACGACCTTAGACTG-3′, reverse: 5′- CAGGTTTGACTTGTCTGAGGTT-3′ and kinase insert domain protein receptor (KDR), forward: 5′- TTTGGCAAATACAACCCTTCAGA-3′, reverse: 5′- GCAGAAGATACTGTCACCACC-3′.

### Western Blotting

For in vivo protein analysis muscle samples were homogenized and processed for Western Blotting as suggested by the manufacturer. Monoclonal anti-VEGF antibodies were purchased from Dako (Glostrup, Denmark).

HUVECs were processed for western blotting as suggested by the manufacturer. Polyclonal anti-VEGF antibody was purchased from Abcam (Cambridge, UK) and analyzed 24 hours after treatment. Polyclonal phoshpo-p44/42 MAPK (ERK1/2) antibody was purchased from Cell Signaling Technology (Massachusetts, USA). Analysis was performed 30 minutes after treatment.

### Cell Culture, VEGF receptor phosphorylation and angiogenesis array

After written informed consent of patients umbilical cords were obtained from Caesarean sections at the Dept. for Gynaecology for isolation of human umbilical vein endothelial cells (HUVECs). Permission was given from the ethics committee of Innsbruck Medical University (No. UN4435). Isolation was performed as described elsewhere [23]. Freshly isolated HUVECs were cultivated in endothelial cell basal medium (CC-3156, Lonza, Walkersville, USA) supplemented with EGM-2 SingleQuots supplements (CC-4176, Lonza). 4×105 cells were suspended per T25 flask 12 h before treatment. Cells used in this experiments all were in passage 5. Two culture flasks were used for each group.

To apply shock waves properly to the cells, the T25 culture flasks were dunked into a water bath [24]. This water bath was built to enable further propagation of shock waves after passing the cell culture as waves would otherwise be reflected at the culture medium to ambient air transition. Reflected waves then would disturb the upcoming ones. In addition, a V-shaped absorber was placed at the back of the bath. The temperature of the water was constantly held at 37°C using a heater triggered by a temperature sensor.

Human phospho-RTK (receptor tyrosine kinase) and human angiogenesis array Kits (R&D, ARY001+ARY007) were used for investigation of receptors and proteins involved in HUVEC signaling after SWT. After stimulation with SWT and incubation for 60 minutes, cells were treated as recommended by the manufacturer for RTK profiler analysis. Angiogenesis profiler was performed according to the manufacturers protocol 24 hours after treatment. In each of the profilers a total of 400 µg protein was used.

For achievement of hypoxic condition HUVECs were placed into a common hypoxic chamber for 16 hours with O_2_<0.5%.

For VEGF receptor 2 inhibition HUVECs were pre-incubated for 1 hour prior to treatment with 100 nM Vandetanib purchased from Selleckchem (Texas, USA).

### Aortic Ring Assay

The aortic ring assay was performed as described [25]. Briefly, thoracic aortas from 12–14 week old C57Bl6 mice (Charles River Laboratories, Wilmington, Ma) were obtained under sterile conditions and cut into 1 mm rings. Aortic rings were incubated with Opti-MEM + GlutaMAX-1 Medium (Gibco, Life Technologies, Grand Island, NY) for 24 hours. Following SWT or sham treatment rings were embedded into collagen matrix containing DMEM Medium (Gibco, Life Technologies, Grand Island, NY) and 1 mg ml^−1^ type I rat tail collagen (Millipore, Billerica, Ma) in 96 well plates. Aortic rings were observed over a period of 7 days. After fixation with 4% paraformaldehyde aortic rings were stained using Rhodamine labeled Griffonia (Bandeiraea) Simplicifolia Lectin I (RL-1102, VectorLabs, Burlingame, CA). DAPI (Life Technologies, Carlsbad, Ca) was used for nuclear counterstaining. Number of rings per group was 6 to 8.

### Immunofluorescence Staining

Immunofluorescence staining was performed as described previously [22]. Briefly, muscle samples were fixed in 4% formaldehyde and subsequently embedded in paraffin. Prior to the staining procedure, heat-mediated antigen retrieval was performed in sodium-citrate buffer (10 mM sodium-citrate, 0,05%Tween 20, pH 6,0) followed by fixation in methanol for 10 min at 4°C. After blocking for 30 min. with 2% BSA in PBS, samples were incubated with monoclonal rat anti-CD31 ( nova, Hamburg, Germany) and rabbit polyclonal anti-alphha smooth muscle actin antibodies (Abcam, Cambridge, UK) over night at 4°C. Alexa Fluor 568 goat anti-rat IgG as well as Alexa Fluor 488 goat anti-rabbit IgG (Life Technologies, Carlsbad, Ca) served as secondary antibodies. DAPI (Life Technologies, Carlsbad, Ca) was used for nuclear counterstaining. Images were analyzed using AxioVision Rel.4.8 software (Carl Zeiss, Oberkochen, Germany). Analyses were performed by a single blinded researcher.

### Laser Doppler Perfusion Imaging (LDPI)

Blood flow measurements were performed pre hind-limb ischemia, directly (day 0), 2 and 4 weeks afterwards by a laser Doppler perfusion image analyzer (Moor Instruments, USA) as previously reported [26]. To minimize data variables attributable to ambient light and temperature mice were kept on a heating plate at 37°C for approximately 10 minutes before measurement in a darkened room. Blood perfusion is expressed as the laser Doppler perfusion image index representing the ratio of left (operated, ischemic leg) to right (not operated, non ischemic leg) limb blood flow. A ratio of 1 prior to surgery indicated equal blood perfusion in both legs.

### Necrosis score

Necrosis score was assessed as described previously [27]. Briefly, mice were investigated at 0, 14 and 28 days post induction of hind limb ischemia and scored with 0 points if no necrosis or defect was observed, with 1 point if skin necrosis was present, with 2 points if below ankle amputation was present and with 3 points if above ankle amputation was observed.

### Statistical Analysis

All results are expressed as mean ± SEM (standard error of the mean). Statistical comparisons between 2 groups were performed by student’s t-test. Continuous variables were either compared with analysis of variance (Bonferroni) after testing for normality of distribution or the Mann–Whitney test. P-values <.05 were considered statistically significant.

## Results

### Increase of angiogenic factors in shock wave treated ischemic muscle

**Figure 1:**
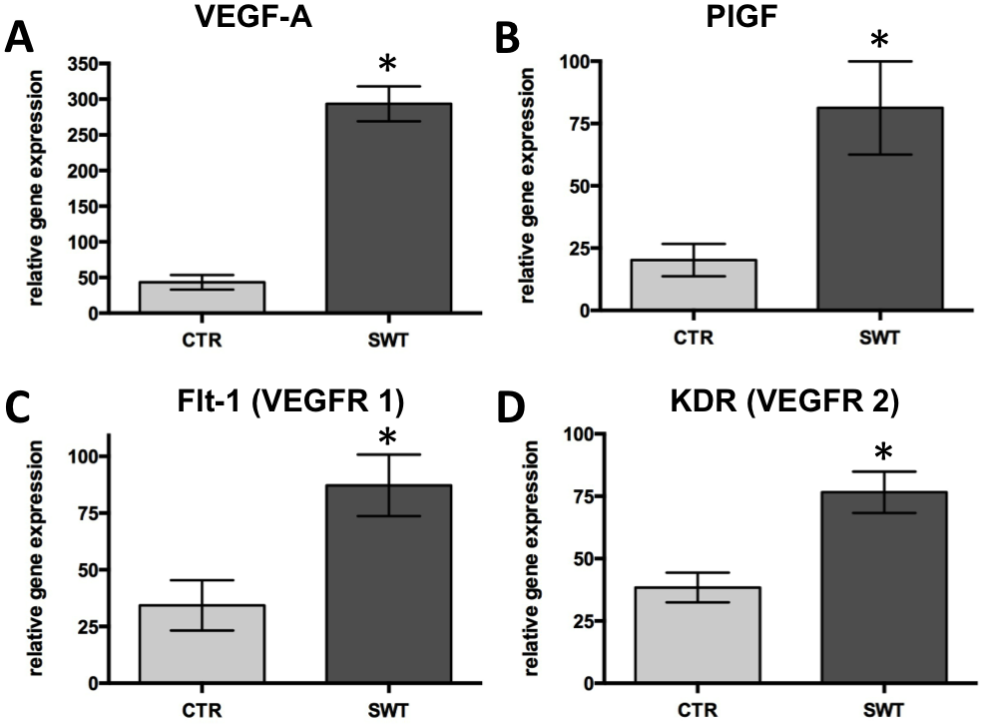
Increase of angiogenic factors in shock wave treated ischemic muscle. Significantly elevated mRNA levels of angiogenic growth factors A, VEGF-A and B, PlGF 72 hours after shock wave treatment (SWT). In line with these findings their main receptors C, Flt-1 and D, KDR were significantly upregulated in the treatment group compared to untreated controls (CTR). *p<.05.

Real-time PCR analyses revealed higher gene expression of angiogenic growth factors VEGF-A (SWT 293.65±20.01 vs. CTR 43.33±9.34, p<0.001) and PlGF (SWT 61.62±15.84 vs. CTR 20.21±5.28, p = 0.048) (**Figure 1A** **+B**), as well as of their receptors Flt-1 (SWT 87.27±11.08 vs. CTR 34.33±10.16, p = 0.009) and KDR (SWT 76.60±6.77 vs. CTR 38.40±5.46, p = 0.006) (**Figure 1C** **+D**) at 72 hours after left femoral artery excision and shock wave treatment.

VEGF is well known to induce angiogenesis hereby forming new capillaries in ischemic tissue. PlGF amplifies the angiogenic activity of VEGF and induces further VEGF release. Moreover, PlGF attracts smooth muscle cells for the coverage of capillaries and therefore forms stable mature arterioles that are crucial for the proposed long-term benefit after shock wave treatment. Indeed, a significantly higher number of arterioles could be found in the SWT group compared to their untreated controls.

**Figure 2:**
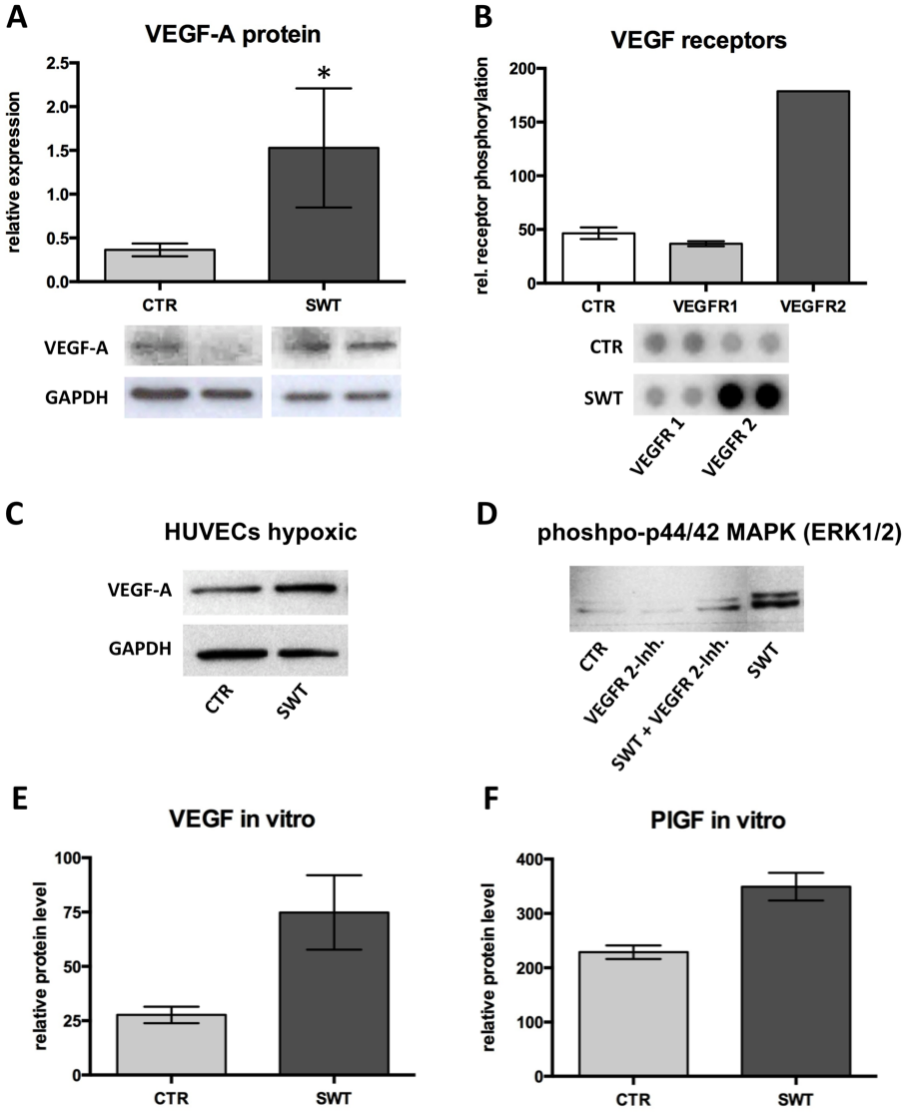
VEGF-A protein expression and stimulation of VEGF receptor 2. A, Pivotal growth factor VEGF-A protein was measured 4 weeks after left femoral artery excision and shock wave treatment by western blot analysis of muscle samples. VEGF-A protein was significantly increased in the treatment group (SWT) compared to untreated controls (CTR). *p<.05. B, Profiler assays were used to investigate the role of receptor tyrosine kinase after shock wave treatment. Results indicate that shock waves stimulate phosphorylation of VEGF receptor 2 whereas there was no effect observed on VEGFR1 phosphorylation. Increase in VEGFR2 phosphorylation is nearly five-fold compared to untreated controls. C, Western blot of HUVECs under hypoxic conditions showing clearly higher protein levels of VEGF-A after shock wave treatment. D, Western blot of phoshpo-p44/42 MAPK (ERK1/2) shows that VEGF signaling is abolished in shock wave treated HUVECs that were pre-incubated with VEGF receptor 2 inhibitor Vandetanib. This finding indicates that shock wave effects are VEGFR2-dependent. E, Relative protein levels of VEGF 24 hours after shock wave treatment of human umbilical vein endothelial cells (HUVECs) as assessed by angiogenesis profiler. F, Relative protein levels of PlGF 24 hours after treatment of HUVECs.

Western blot analysis consequently showed significant up-regulation of VEGF-A protein (SWT 1.53±0.56 vs. CTR 0.36±0.07, p = 0.03) 72 hours after shock wave treatment compared to untreated controls (**Figure 2A**).

### Phosphorylation of VEGF receptors and angiogenic protein expression in endothelial cells

Having shown the significant up-regulation of VEGF on mRNA and protein levels we aimed to elucidate whether shock wave treatment induces angiogenesis also by direct stimulation of endothelial cells. Therefore, we investigated shock wave induced signaling in human umbilical vein endothelial cells (HUVECs) using receptor tyrosine kinase (RTK) profiler assays. This assay reveals activated receptors by means of phosphorylation. Quantification of relative VEGFR phosphorylation showed a significant, nearly five-fold activation of VEGF receptor 2 (VEGFR) 60 minutes after treatment whereas no effect could be observed on VEGF receptor 1 (VEGFR1, SWT 36.72 vs. CTR 55.71; VEGFR2, SWT 178.72 vs. CTR 37.17) (**Figure 2B**).

This indicates a direct effect of SWT on phosphorylation of VEGFR 2, which is known to be the pivotal VEGF receptor for induction of angiogenesis. This confirms our findings of increased numbers of vessels. Thereby phosphorylation of VEGFR 2 may represent the main mechanism of action of shock waves to ischemic muscle. However, treated HUVECs were under common normoxic cell culture conditions. Therefore, we additionally performed Western blots of VEGF in HUVECs that were incubated under hypoxic conditions. Shock wave treated cells showed an increased amount of VEGF protein compared to untreated controls and confirming the findings of in vivo ischemia (**Figure 2** **C**).

In order to verify whether the effect of shock waves is actually VEGF receptor 2 dependent we pre-incubated HUVECs with the VEGFR 2 inhibitor Vandetanib. Indeed, the effect of shock waves was almost completely abolished as shown by Western blot analysis of pivotal VEGF signaling kinase phoshpo-p44/42 MAPK (ERK1/2) (**Figure 2D**)**.**

To further elucidate whether shock waves do as well have a direct effect on growth factor release from endothelial cells we performed an angiogenesis array. It confirmed an increase of VEGF and PlGF protein expression in SW treated endothelial cells compared to untreated controls (VEGF: SWT 74.82±9.89 vs. CTR 27.67±2.2; PlGF: SWT 349.26±14.67 vs. CTR 228.74±7.18) (**Figure 2E** **+F**). These data indicate that growth factors may mechanically get released from extracellular matrix where they are bound to heparansulfate proteoglycans. However, further experiments are necessary to proof this hypothesis.

### Aortic ring assay

**Figure 3:**
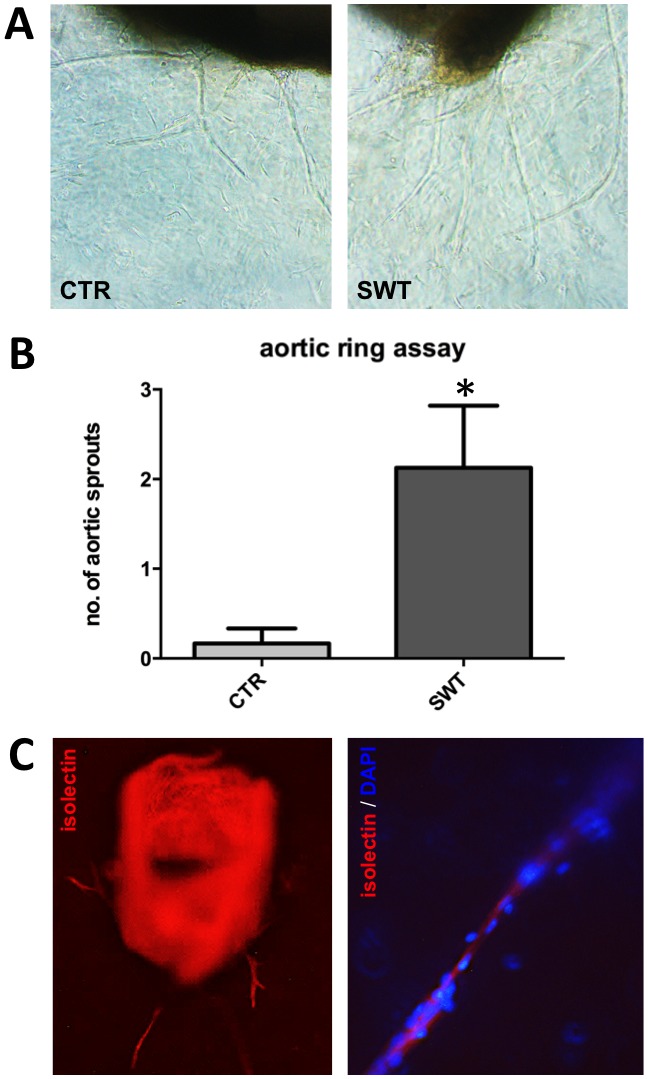
Higher numbers of capillary sprouts from aortic rings in the treatment group. A, Representative pictures from light microscope of untreated (CTR) and shock wave treated (SWT) mouse aortic rings depicting higher numbers of sprouts in the treatment group 7 days following SWT. B, Quantification of sprouts revealed a significant increase in the treatment group, *p<.05, n = 6–8 per group. C, Immunofluorescence confirmed that sprouts consist of endothelial cells (red: lectin endothelial staining, blue: DAPI nucleus counterstaining).

In order to proof whether the angiogenic stimulus is mainly derived from endothelium, but not myocytes, we performed an aortic ring assay. Significantly higher numbers of vessel sprouts from mouse aortic rings could be found in the shock wave treated group compared to untreated aortic rings (SWT 2.13±0.69 vs. CTR 0.17±0.17, p = 0.034) (**Figure 3A** **+B**). We therefore propose that the increase in microvascular density in ischemic muscles is mainly due to an activation of endothelium. To make sure that sprouts seen under the light microscope actually represent capillaries we performed lectin staining for endothelial cells. Thereby we have been able to show that shock wave induced sprouts from aortic rings indeed are formed by endothelial cells (**Figure 3C**).

### Increase of capillary and arteriole density in ischemic muscle

**Figure 4:**
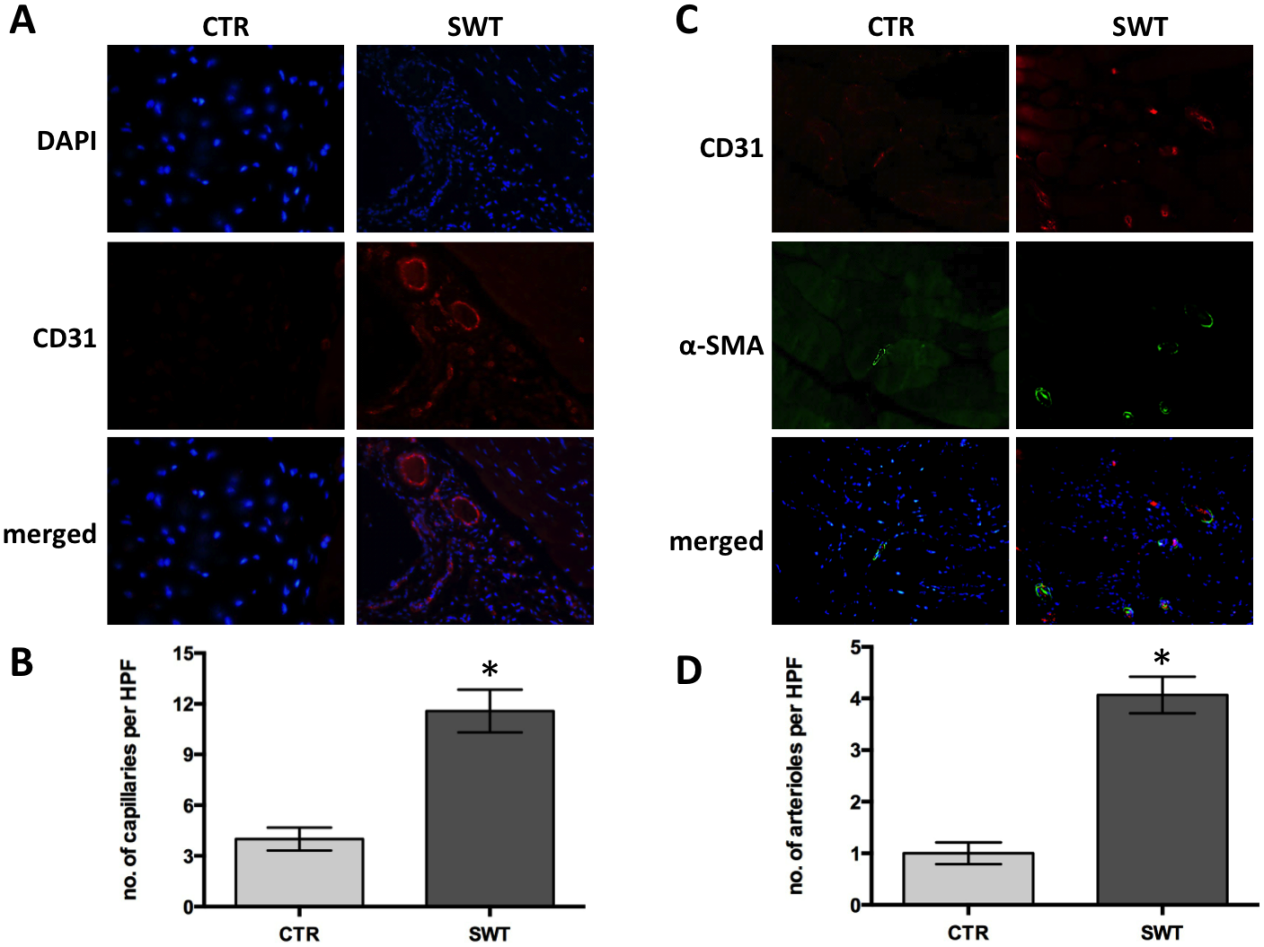
Increase of capillary and arteriole density in ischemic muscle. A, Representative views of immunofluorescence stainings for DAPI (cell nuclei) and CD31 (endothelial cells). Magnification x400; B, Increase of capillary density in treated ischemic muscle. Immunofluorescence staining for CD31-positive cells revealed significantly higher numbers of capillaries per high power field (HPF) 4 weeks after shock wave therapy (SWT). *p<.05. C, Representative views of immunofluorescence stainings for arterioles (CD31– endothelial cells, alpha-SMA – smooth muscle cells). Magnification x200; D, Quantification of arteriole staining revealed significantly more arterioles per HPF in shock wave treated muscle. *p<.05.

Quantitative immunofluorescence was performed to evaluate vessel formation 4 weeks after ischemia and shock wave treatment. It revealed an increased number of CD31 positive capillaries per high-power field (SWT 12±2 vs. CTR 4±1, p<0.001), being a correlate for a higher micro-vascular density in the shock wave group compared to controls (**Figure 4A** **+B**).

A co-staining for CD31 (endothelial cell staining) and alpha smooth muscle actin (staining for smooth muscle cells) was performed to evaluate whether shock wave treatment also induces the formation of mature arterioles that in contrast to capillaries are covered by a smooth muscle layer. Significantly higher numbers of arterioles per high power field could be detected in the SWT group compared to untreated controls (SWT 4±1 vs. CTR 1±0.5, p<0.001) (**Figure 4C** **+D**). This finding 4 weeks after shock wave treatment indicates a stable long-term result of angiogenesis in ischemic tissue.

### Improvement of blood perfusion and limb necrosis

**Figure 5:**
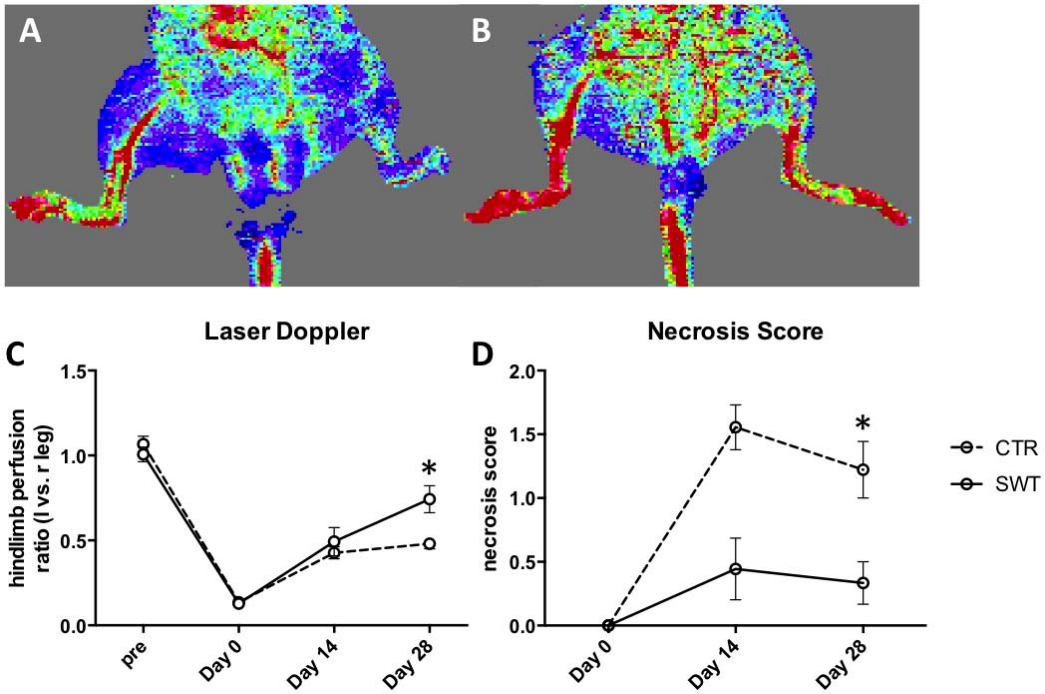
Improvement of blood perfusion and limb necrosis. Blood perfusion of hind limbs measured by laser Doppler perfusion imaging pre hind-limb ischemia, directly afterwards (day 0), 2 and 4 weeks after left femoral artery excision and shock wave therapy. A, representative picture of untreated control (CTR) after 4 weeks; B, representative picture of shock wave treated animal (SWT) after 4 weeks; C, Quantification of measurements expressed as ratio of left leg (operated, ischemic) to right leg (non-operated, non-ischemic) revealing significant improvement after 4 weeks in the treatment group compared to untreated controls. *p<.05. D, Necrosis score was assessed at baseline, after 14 and 28 days. Although untreated controls show a notable self-regeneration, necrosis score shows significant improvement in SWT mice after 28 days. *p<.05.

Blood perfusion of hind limbs was measured by laser Doppler perfusion imaging pre hind-limb ischemia and treatment, directly afterwards (day 0) as well as 2 and 4 weeks after femoral artery excision and shock wave treatment (**Figure 5A** **+B**). Although untreated control animals show a notable self-regeneration potential, significant improvement could be observed after 4 weeks in the treatment group (SWT 0.74±0.01 vs. CTR 0.48±0.01, p = 0.021) (**Figure 5C**). Data is expressed as ratio left (operated, ischemic) to right (control, non ischemic) leg. To strengthen these findings animals were furthermore investigated for a clinical outcome parameter by necrosis score assessment examined 0, 14 and 28 days after therapy. Necrosis score showed a significant improvement after 4 weeks in the treatment group compared to untreated controls (SWT 0.33±0.2 vs. CTR 1.11±0.25, p = 0.004) (**Figure 5D**).

Improvement of blood perfusion as well as of limb necrosis 4 weeks after shock wave treatment shows the clinical benefit of this new treatment option for acute muscle ischemia.

## Discussion

Shock wave therapy is developing for a large variety of indications, including acute and chronic soft tissue wounds [19]–[21]. In chronic myocardial infarction and limb ischemia it has been shown to induce angiogenesis and thereby improvement of clinical outcome [13], [16]. Results in soft tissue wounds seem to be independent from the time point of treatment [21].

The present experiments were intended to proof the induction of angiogenesis in the treatment of acute hind-limb ischemia and to evaluate a stable long-term benefit resulting in improvement of blood perfusion.

Shock wave treatment as an adjunct to state-of-the-art revascularization in acute myocardial infarction or limb ischemia could dramatically improve outcome and quality of life if the effects were comparable to those seen in the chronic setting.

We therefore performed a hind limb ischemia model in mice by excision of the left femoral artery. Changes in gene expression of the main angiogenic factors were measured 72 hours after shock wave treatment. Comparable to results from the treatment of chronic myocardium and muscle ischemia we found an up-regulation of pivotal growth factors VEGF and PlGF mRNA [13], [16]. Moreover a significant increase of VEGF protein could be observed 4 weeks following SWT. VEGF is well known to induce angiogenesis hereby forming new capillaries in ischemic tissue. PlGF amplifies the angiogenic activity of VEGF and induces further VEGF release. Additionally, PlGF attracts smooth muscle cells for the coverage of capillaries and therefore forms stable mature arterioles that are crucial for the proposed long-term benefit after shock wave treatment.

As an underlying mechanism of action of shock wave treatment we revealed a direct effect to VEGF receptor 2 phosphorylation in endothelial cells. Using a VEGFR 2 inhibitor effects on VEGF signaling were almost completely abolished. This confirmed that the shock wave mechanism is VEGFR2 dependent. VEGFR 2 is known to be the pivotal VEGF receptor for the induction of angiogenesis. Indeed, as a consequence we found increased levels of VEGF and PlGF protein after shock wave treatment in endothelial cells. In order to clarify whether endothelial cells may be the main source of angiogenesis induction following SWT we performed an aortic ring assay. It revealed that the induction of capillary sprouting from mouse aorta ex-vivo is possible. Thereby this experiment proofs that myocytes are not a prerequisite source of angiogenesis induction.

In line with these findings quantitative immunofluorescence from muscle samples revealed significantly higher numbers of capillaries as well as arterioles covered with smooth muscle cells in treated ischemic muscle compared to untreated controls 4 weeks after induction of ischemia.

This higher micro-vascular density may be the main reason for the restoration of blood perfusion as shown by laser Doppler perfusion imaging. Increased blood perfusion resulted in less necrosis in the treatment group.

Still today results of surgical and interventional revascularization in limb or myocardial ischemia are limited by non-viable and hibernating myocardium or skeletal muscle due to diffuse artery disease of small vessels [28]–[30].

Therefore, therapies that induce angiogenesis and muscle regeneration adjunctive to state-of-the-art revascularization might improve survival and functional outcome in patients suffering from acute ischemia. Numerous modalities, including gene and cell-based approaches are currently tested in this respect [31]–[34]. A novel approach to promote angiogenesis is low energy shock wave treatment [12], [13], [16]–[21].

Biological induction of neovascularization in addition to surgical or interventional revascularization could dramatically improve outcome by recruitment of larger areas of ischemic tissue, such as hibernating myocardium [35]–[37].

The results of the present study therefore suggest that low energy shock wave treatment could develop a feasible adjunct to surgical and interventional revascularization in the setting of acute myocardial or limb ischemia. The translation of our findings to human application seems close as the treatment of soft tissue wounds already is clinically implemented [8], [19]. Moreover, the treatment of chronic myocardial ischemia has already been studied successfully in humans [10]–[12].
